# “Why” or “How”: The Effect of Concrete Versus Abstract Processing on Intrusive Memories Following Analogue Trauma^☆^^☆☆^

**DOI:** 10.1016/j.beth.2016.02.004

**Published:** 2016-05

**Authors:** Rachel White, Jennifer Wild

**Affiliations:** Institute of Psychiatry, King’s College London; University of Oxford

**Keywords:** trauma, intrusive memories, processing mode, abstract, concrete

## Abstract

Emergency service workers, military personnel, and journalists working in conflict zones are regularly exposed to trauma as part of their jobs and suffer higher rates of posttraumatic stress compared with the general population. These individuals often know that they will be exposed to trauma and therefore have the opportunity to adopt potentially protective cognitive strategies. One cognitive strategy linked to better mood and recovery from upsetting events is concrete information processing. Conversely, abstract information processing is linked to the development of anxiety and depression. We trained 50 healthy participants to apply an abstract or concrete mode of processing to six traumatic film clips and to apply this mode of processing to a posttraining traumatic film. Intrusive memories of the films were recorded for 1 week and the Impact of Events Scale–Revised (IES-R; Weiss & Marmar, 1997) was completed at 1-week follow-up. As predicted, participants in the concrete condition reported significantly fewer intrusive memories in response to the films and had lower IES-R scores compared with those in the abstract condition. They also showed reduced emotional reactivity to the posttraining film. Self-reported proneness to intrusive memories in everyday life was significantly correlated with intrusive memories of the films, whereas trait rumination, trait dissociation, and sleep difficulties were not. Findings suggest that training individuals to adopt a concrete mode of information processing during analogue trauma may protect against the development of intrusive memories.

## Highlights

• We compared abstract versus concrete information processing of traumatic stimuli
• Concrete processing led to fewer intrusive memories than abstract processing
• Concrete processing led to reduced severity of PTSD symptoms than abstract processing
• Concrete processing led to lower emotional reactivity to a subsequent stimulus relative to abstract processing
• Proneness to intrusive memories was associated with outcome

Cognitive Models of Posttraumatic Stress Disorder (PTSD; e.g., Brewin et al., 1996, Ehlers and Clark, 2000) emphasize peritraumatic processing as an important factor influencing the development of PTSD. According to these models, dysfunctional information processing may disrupt the formation of a coherent narrative of the event, giving rise to intrusive memories and associated distress. In the wider literature, a distinction is made between abstract and concrete modes of information processing. Concrete processing focuses on “how” an event is happening, on direct experience, and means to desired ends (e.g., steps needed to achieve a goal), whereas abstract processing is characterized by generalized thoughts conveying overall meaning as well as “why?” and “what if?” questions with no obvious answer (Watkins, 2008). Processes such as worry and rumination, which are associated with a range of disorders including PTSD, are characterized by an abstract style of processing (Behar et al., 2012, Goldwin and Behar, 2012, Stöber et al., 2002, Watkins and Moulds, 2007). According to theories of worry, it is the abstract-verbal (as opposed to concrete-imagery-based) nature of worry that accounts for its maladaptive effects. It is hypothesized that this thinking style inhibits emotional processing of fearful imagery, strengthening anxious meanings and interfering with problem solving (Behar et al., 2012, Borkovec et al., 2004, Stöber, 1998). Recent research found that ambulance workers who adopted more abstract “why?” thoughts during exposure to trauma reported poorer levels of coping, suggesting that abstract thinking during trauma may be unhelpful (Shepherd & Wild, 2014). In this study, we investigated the link between abstract and concrete modes of processing during analogue trauma and the subsequent development of intrusive memories.

Accumulating evidence suggests that training individuals to process events (either during or afterwards) in a concrete relative to an abstract way increases resilience to these events. Watkins, Moberly, and Moulds (2008) trained participants to think about ambiguous scenarios in a concrete or abstract way, and found that concrete training led to reduced despondency in response to a subsequent failure experience compared with abstract training. In an earlier study, Watkins (2004) instructed healthy participants to think about an induced failure experience afterwards in an abstract or concrete way. Those who were instructed to think about the failure experience in a concrete way experienced fewer intrusive memories of it compared with those who had thought about it in an abstract way. Watkins, Baeyens, and Read (2009) found that concreteness training also reduced depressive symptoms in dysphoric individuals and suggested that concreteness training could be used as a guided self-help intervention for individuals with mild to moderate depressive symptoms.

Ehring, Szeimies, and Schaffrick (2009) investigated whether post-event abstract processing could account for the maladaptive effects of trauma-related rumination using the trauma film paradigm. This paradigm induces analogue posttraumatic stress symptoms (e.g., intrusive memories and distress) by exposing participants to analogue trauma (i.e., traumatic films), and allows for the manipulation of variables that might increase or decrease symptoms (see Holmes & Bourne, 2008, for a review). Ehring and colleagues exposed healthy participants to a traumatic film depicting the aftermath of road traffic accidents, and then randomly assigned them to one of three conditions (abstract rumination, concrete thinking, or a distraction control) for 10 minutes. In the abstract condition, participants were asked to dwell on sentences containing abstract thoughts about the footage (e.g., “Why do so many accidents have to happen?”), whereas in the concrete condition, they were asked to read sentences containing concrete thoughts (e.g., “What are the different reasons for accidents happening?”). In the distraction condition, they were asked to read unrelated, neutral sentences. Abstract rumination led to significantly longer duration of negative mood and arousal following the film than did concrete thinking and distraction. No significant differences in number, vividness, or distress of intrusive memories during a 2-minute follow-up period were found between the abstract and concrete conditions and, unexpectedly, those in the distraction condition showed the highest number of intrusions. Participants in the concrete condition reported significantly fewer intrusive memories than those in the distraction condition, whereas there was no significant difference in number of intrusive memories between those in the abstract and distraction conditions.

Studies adopting the trauma film paradigm can inform our understanding of how to modify peritraumatic processes to potentially influence the development of symptoms associated with PTSD. Using such a paradigm, Schartau, Dalgleish, and Dunn (2009) investigated whether training individuals to apply adaptive appraisals to a series of distressing films would reduce their emotional reactivity to a subsequent traumatic film. They trained healthy individuals to apply these appraisals to a series of distressing films or to watch the films without trying to regulate their emotions in any way. The appraisals were based on themes of seeing “the bigger picture,” such as “bad things happen,” “silver lining,” “broader perspective,” and “time heals.” Their emotional reactions to test films pre- and post-training were compared with controls’. Participants who underwent appraisal training reported less negative emotion (distress and horror) and exhibited a greater reduction in psychophysiological responses from pre- to posttraining compared with the control group.

The effect of abstract versus concrete peritraumatic processing has yet to be investigated in relation to intrusive memory development. In addition to processing mode, a number of other individual difference variables may affect vulnerability to developing intrusive memories. Both trait rumination and dissociation have been linked to the development of intrusive memories and PTSD (Murray, 1997, Murray et al., 2002, Nolen-Hoeksema and Morrow, 1991). Sleep difficulties have also been implicated in the development and maintenance of intrusive memories. Mellman, David, Kulick-Bell, Hebding, and Nolan (1995) found that greater pretrauma sleep difficulties predicted the development of PTSD, although these were rated retrospectively and therefore may have been influenced by recall bias. Finally, another factor that has been linked to intrusive memories is individual proneness to intrusive memories. Davies and Clark (1998) found that individuals who reported a higher tendency to experience intrusive memories in their everyday lives experienced more intrusive memories in response to a traumatic film.

Using a trauma film paradigm, we investigated the effect of training individuals to apply either an abstract or concrete processing mode during exposure to an analogue trauma on the subsequent development of intrusive memories. We also investigated the effect of these training procedures on emotional reactivity to a subsequent stressor and the relationship between potential vulnerability factors and the development of intrusions. We hypothesized that: (a) compared with abstract training, concrete training while watching a series of traumatic films would lead to fewer intrusive memories of the films and lower scores on a measure of PTSD severity at 1-week follow-up; (b) compared with abstract training, concrete training would lead to reduced emotional reactivity to a subsequent stressor; and (c) individual vulnerability factors previously linked to PTSD (trait rumination, trait dissociation, preexisting sleep difficulties, and proneness to intrusive memories) would be associated with the development of intrusive memories and scores on the measure of PTSD symptom severity. Finally, since low mood is associated with intrusions (Davies & Clark, 1998) and exposure to analogue trauma can reduce mood (Ehring et al., 2009, Zetsche et al., 2009), we investigated whether reductions in mood during the experimental session were linked to the development of intrusions.

## Method

### Participants

A power analysis based on the effect sizes from a similar study by Schartau et al. (2009) was used to determine the sample size. Results showed that a sample size of 25 in each condition would have 80% power to detect a significant difference in mean change scores between the abstract and concrete conditions using a two-group *t* test with a two-tailed .05 significance level. Therefore, we concluded that a total of 50 participants would be needed.

Participants over the age of 18 were recruited from King’s College London through an email circular. We excluded participants who self-reported a mental health problem, scored 10 or higher on a standardized measure of depression, or scored 33 or higher on a standardized measure of posttraumatic stress. We excluded these individuals for three reasons: (a) as an ethical measure to reduce distress in already vulnerable individuals; (b) to increase homogeneity of participants across the two conditions; and (c) to replicate the samples of studies that have previously used the trauma film paradigm (e.g., Ehring et al., 2009, Schartau et al., 2009). A total of 2 participants were excluded for scoring above cutoff on the screening measures, and 1 participant was excluded for failure to follow the instructions during the posttraining test film. This left 50 participants: 26 were randomly assigned to the abstract condition (15 female, *M*_age_ = 27.15, *SD* = 9.11), and 24 to the concrete condition (13 female, *M*_age_ = 24.71, *SD* = 5.35). Conditions were comparable on age, *t*(48) = 1.15, *p* = .258) and sex, χ^2^(1) = 0.06, *p* = .802. Ethical approval for the study was granted by the Psychiatry, Nursing and Midwifery Research Ethics Committee at King’s College London.

### Measures

#### Demographic Questionnaire (Unpublished)

A brief demographic questionnaire was included to collect relevant demographic information to ensure equivalence of conditions on age, gender, and driving frequency (since most of the films contained exposure to RTA footage).

#### Patient Health Questionnaire–9 (Kroenke, Spitzer, & Williams, 2001)

Depression was assessed using the Patient Health Questionnaire–9 (PHQ-9; Kroenke, Spitzer, & Williams, 2001), a brief depression severity measure consisting of 9 items. Scores range from 0 to 27, with higher scores indicating greater depression severity. This measure has demonstrated high internal consistency (α = .86–.89), good test–retest reliability (*r* = .84), and correlates significantly with other standardized measures of depression. The recommended cutoff of ≥ 10 (Kroenke et al., 2001) was used to indicate major depression in this study. Internal consistency for the current study was modest (Cronbach’s α = .50).

#### Impact of Event Scale–Revised (Weiss & Marmar, 1997)

Posttraumatic stress symptoms (both prior to participation and at 1-week follow-up) were assessed using the Impact of Event Scale–Revised (IES-R; Weiss & Marmar, 1997), a widely used measure of PTSD severity, consisting of 22 items. Scores range from 0 to 88, with higher scores indicating greater severity of PTSD symptoms. The scale has shown high internal consistency (α = .96; Creamer, Bell, & Failla, 2003) and test–retest reliability coefficients that range from *r* = .51 to .94 for the individual subscales (Weiss & Marmar, 1997). Concurrent validity has been documented, with the scale being highly correlated (*r* = .84) with the Posttraumatic Checklist (Blanchard, Jones-Alexander, Buckley, & Forneris, 1996), an established measure of PTSD (Creamer et al., 2003). We used a cutoff of ≥ 33 to indicate clinically significant levels of PTSD, as recommended by Creamer et al. (2003). High internal consistency was found for the current sample both for preexisting symptom levels (α = .87) and at 1-week follow-up (α = .89).

#### Trauma Screener (Unpublished)

Participants also completed the Trauma Screener (unpublished), a self-report inventory of prior exposure to traumatic events, which has been used in previous studies (e.g., Ehlers, Mayou, & Bryant, 1998) and is derived from the trauma checklist included in the Clinician-Administered PTSD Scale (Blake et al., 1990). It includes a checklist of 16 events such as serious traffic accidents, serious other accidents (e.g., fire or explosion), sexual or nonsexual assault, and others. It was used to establish an index traumatic event for completing the IES-R and to ensure that the event was one in which the person “experienced, witnessed or was confronted with an event or events that involved actual or threatened death or serious physical injury, or a threat to the physical integrity of self or others” (*Diagnostic and Statistical Manual* [DSM-IV]; APA, 1994).

#### State–Trait Anxiety Inventory–Trait Version (Spielberger, Gorsuch, Lushene, Vagg, & Jacobs, 1983)

The State–Trait Anxiety Inventory–Trait Version (STAI-T) was administered to assess equivalence of conditions in proneness to anxiety. This is a widely used self-report measure that assesses tendency to feel anxious in response to stressful situations (Spielberger et al., 1983). It contains 20 items, with scores ranging from 20 to 80, and higher scores indicating higher levels of trait anxiety. It has demonstrated high internal consistency (median α = .90), acceptable test–retest reliability ranging from *r* = .73 to *r* = .86 in nonclinical samples, and it correlates with other trait anxiety measures (Spielberger et al., 1983). Internal consistency for the current study was high (α = .87).

#### Intrusions Diary (Unpublished)

Number of intrusive memories experienced in the week following the experiment was measured using an intrusions diary, a standard way of assessing the frequency of intrusions (Holmes & Bourne, 2008). Participants were asked to record daily the number of times they had experienced spontaneously occurring intrusive memories of any of the films. For each intrusion they had, they were asked to record which film it referred to (to check that intrusions corresponded to films viewed in the study).

#### Perseverative Thinking Questionnaire (Ehring et al., 2010)

Trait rumination was measured using the Perseverative Thinking Questionnaire (PTQ; Ehring et al., 2010), a content-independent self-report measure of repetitive negative thinking (including rumination). It contains 15 items, with scores ranging from 0 to 60, with higher scores indicating a greater tendency toward repetitive negative thinking. Excellent internal consistency (α = 0.94–0.95) but limited test–retest reliability (*r* = 0.69) have been reported. It demonstrates convergent validity, correlating significantly with standard measures of rumination and worry (ranging from *r* = .48 to *r* = .72; Ehring et al., 2010). It also shows concurrent validity, with significant and moderate correlations found with severity of anxiety (*r* = .64) and depressive (*r* = .54–.58) symptoms (Ehring et al., 2010). Internal consistency was high for the current sample (α = .94).

#### Trait Dissociation Questionnaire–Short Version (TDQs; Murray, 1997)

Trait dissociation was measured using the Trait Dissociation Questionnaire–Short Version (TDQs; Murray, 1997), a measure of trait disposition to dissociate. It contains 11 items, with scores ranging from 0 to 55, with higher scores reflecting greater trait dissociation. The measure has been validated using an outpatient sample and correlates highly with the original TDQ (*r* = 0.94), which has been shown to predict intrusive memories in a student population. It evidences high internal consistency (α = 0.86) but limited test–retest reliability (*r* = 0.56; Murray, 1997). Internal consistency for the current sample was acceptable (α = .74).

#### Proneness to Intrusive Memories Scale (PIMS; Unpublished)

To assess individual proneness to intrusive memories, we administered a 5-point scale asking participants to rate the frequency with which they tended to experience intrusive memories of stressful or unpleasant events ranging from 0 (*not at all*) to 5 (*five times a week or more*).

#### Insomnia Severity Index (ISI; Morin, Barlow, & Dement, 1993)

To assess preexperiment sleep difficulties, we administered the Insomnia Severity Index (ISI; Morin, Barlow, & Dement, 1993), a brief 7-item screening measure for insomnia severity over the previous 2 weeks. Total scores range from 0 to 28, with higher scores indicating higher severity of insomnia. The measure has been validated on younger and older populations attending a sleep clinic or receiving treatment for insomnia, and has demonstrated adequate internal consistency (α = 0.74–0.78). It has also demonstrated concurrent validity, correlating significantly with other measures of insomnia (sleep diaries, polysomnography, and informant and clinician reports), and sensitivity in detecting change in perceived sleep difficulties following treatment (Bastien, Vallières, & Morin, 2001). Internal consistency was high for the current sample (α = .86).

### Procedure

Informed consent was obtained for all participants before taking part in the study. Participants completed the pretraining measures (PTQ, TDQs, PIMS, and ISI) and were randomly allocated to the abstract or concrete training condition to complete the film task. They were told that this involved watching a series of film clips of real-life traumatic footage, which they would be asked to watch in a particular way according to some instructions they would be given. The film task was adapted from Schartau et al. (2009). The two test films (shown pre- and posttraining) contained real-life footage of the aftermath of road traffic accidents (RTAs) and showed emergency service personnel working to extract trapped victims, severely injured individuals in distress, and dead bodies being moved. These were selected from a series of films containing footage of RTAs compiled by Steil (1997), which has been used in previous studies (e.g., Holmes et al., 2004, Murray, 1997, Steil, 1997). The series of films was piloted on a small sample of student volunteers (*N* = 10) from King’s College London who met the study’s eligibility criteria, to establish which two films evoked the most distress and hence would best serve as a pre- and posttraining test films. The two films selected were rated as the most distressing (> 55 on a 0–100 scale) and were comparable in the level of distress they evoked (pretraining film: *M* = 55.30, *SD* = 27.81; posttraining film: *M* = 59.70, *SD* = 23.45; *t*(9) = 1.346, *p* = .211). Three additional RTA films were selected from this series as practice films. The remaining three practice films were borrowed with permission from Schartau et al. (2009) and included non-RTA scenes of violence involving animals and humans. We selected both RTA and non-RTA films so that participants could practice on a range of stimuli. Films ranged from 21 to 137 seconds (*M* = 89.00, *SD* = 35.88). Each film contained a short introduction at the beginning explaining what each depicted.

**Figure 1:**
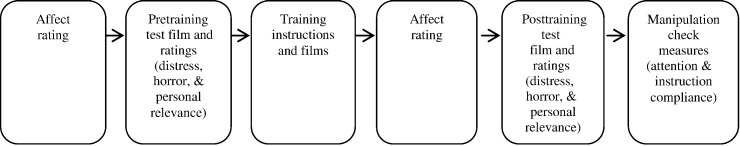
Overview of film task.

Films were shown on a 15-in. computer screen and the film task followed six steps (see Figure 1). First, participants were asked to rate their current affect on a scale ranging from 0 (*extremely negative*) to 100 (*extremely positive*). Second, they were shown the pretraining test film and asked to simply watch it as they would normally, before rating their emotional reactions to it. Emotional reactivity was indexed by two scales ranging from 0 to 100, which measured distress (ranging from *no distress* to *extreme distress*) and horror (ranging from *no horror* to *extreme horror*). Distress and horror were chosen as they were the target emotions used by Schartau et al. (2009), who demonstrated an effect of appraisal training on these emotions in reaction to distressing films. They also noted that generalized emotion terms (e.g., distress and negative emotion) have been demonstrated as reliable indices of emotion change in response to experimental manipulations (Richards & Gross, 2000) and bear similarity to the Subjective Units of Distress measure, which is often used in clinical research and practice (e.g., Dalgleish & Yiend, 2006), as well as being emotions historically elicited by traumatic experiences. Participants also rated the degree of personal relevance of the pretraining test film. They were asked, “How much personal relevance did this film have for you?” with the scale ranging from 0 (*none*) to 100 (*extreme*). This was included to assess any differences between the conditions in the extent to which the films were personally relevant.

Third, participants were given instructions for how to watch the subsequent films and shown the six practice films. Instructions were presented verbally at first, as well as being presented on the computer screen before each practice film. After each film, the word “relax” appeared on the screen for 5 seconds, which aimed to minimize any accumulative effect of the training phase on mood. Participants in the abstract condition received the following instructions: “When watching the films, please focus on: (1) Why these sorts of things happen; (2) What it means about the world; (3) What it means for the people involved; (4) What if this were to happen to you, or someone in your family?” For participants in the concrete condition, the instructions were as follows: “When watching the films, please focus on: (1) The specific and objective details of the event, for example, what you can see, what you can hear; (2) The sequence of events as they are unfolding; (3) What needs to happen step by step from here.” Following Schartau et al. (2009), before the first practice film, participants were given examples of how they might apply the assigned processing mode to it, before practicing applying it on their own. For the remaining films, examples were not given beforehand although after each of the first three films, participants were asked to give examples of their thoughts while watching the film so that the investigator could be sure that they understood the instructions and that their thoughts reflected their assigned processing mode. Where participants were clearly not applying the required mode of processing or their feedback reflected thoughts that were inconsistent with that mode of processing, the instructions were repeated with further clarification and examples where necessary. For the remaining three films, participants were simply asked whether they thought they had successfully applied the required processing mode and prompted to continue doing so.

Fourth, participants were asked to rate their affect for the second time on the scale ranging from 0 (*extremely negative*) to 100 (*extremely positive*).^2^ Fifth, participants were shown the posttraining test film with instructions presented onscreen telling them to watch it in the way that they had been practicing so far, followed by the assessment of emotional reactivity (i.e., self-report ratings of distress and horror in response to the final film) and personal relevance ratings. Sixth, a manipulation check was carried out whereby participants were asked to rate their level of adherence to the instructions on a scale, which asked: “To what extent did you watch the film according to the instructions given to you?” with responses ranging from 0 (*none of the time*) to 100 (*all of the time*). In addition, their level of attention to the film was assessed by asking: “To what extent did you pay attention to the film?” with a scale of responses ranging from 0 (*none of the time*) to 100 (*all of the time*). Participants were excluded if their level of adherence or attention was less than 50%, which resulted in one participant being excluded from the analysis.

Afterwards, participants were given the intrusions diary and asked to record any spontaneous intrusive memories of any of the scenes they saw during the films for the following week. The experimenter checked that participants were not showing significant signs of distress before leaving the session and provided participants with one of the researcher’s contact details should they wish to discuss their experience of the study. Participants posted the diary back 1 week later following an email prompt, which contained a link to an online version of the IES-R that they were asked to complete. They were also asked to rate to what extent they completed the diary reliably and accurately on a 5-point scale, with responses ranging from 0 (*never*) to 4 (*all of the time*). Participants were given a payment of £15 as compensation for their time.

## Results

### Premanipulation Group Differences

Table 1 shows descriptive statistics for demographic and baseline variables by condition. Independent samples *t* tests revealed no differences between the conditions in levels of depression, preexisting PTSD symptoms, number of previous traumas, trait anxiety, trait rumination, trait dissociation, sleep difficulties, proneness to intrusive memories, baseline affect, personal relevance ratings, or emotional reactivity ratings for the pretraining film (all *p*s > .05). A chi-squared test revealed that conditions were also comparable on driving frequency, χ^2^(1) = 0.24, *p* = .877.

### Instruction and Diary Compliance

Participants generally reported paying attention to the posttraining test film (*M* = 92.10, *SD* = 10.84) and adhering to the instructions (*M* = 85.90, *SD* = 10.43), and there were no differences between the conditions on these measures (attention: *t*[48] = 0.02, *p* = .983; adherence: *t*[48] = 0.31, *p* = .757). Participants generally reported completing the diary reliably and accurately most of the time (*M* = 3.79, *SD* = 0.41) and there were no between-group differences, *t*(48) = 0.63, *p* = .534.

### Effect of Processing Mode Training on Affect

**Figure 2:**
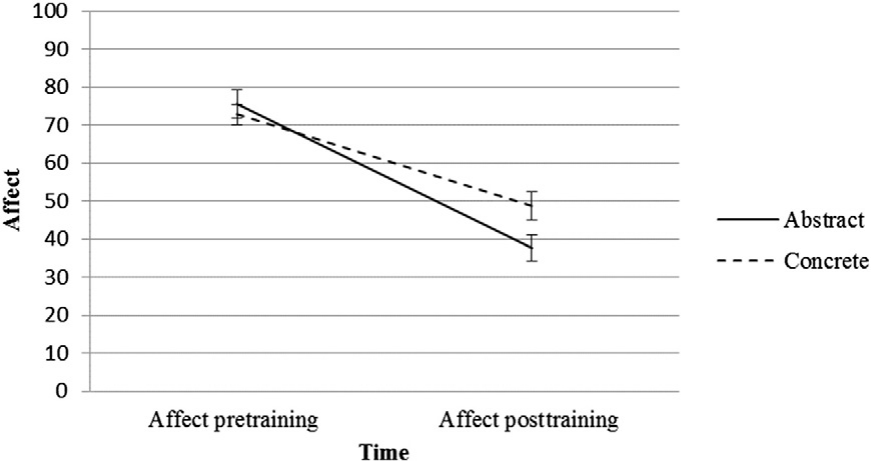
Mean change in ratings of affect (with standard errors) from pre- to posttraining by condition.

To investigate the effect of training on affect following the practice films, we conducted a 2 (Condition: abstract, concrete) × 2 (Time: pretraining, posttraining) mixed model analysis of variance (ANOVA), with Condition as the between-subjects factor and Time as the repeated measures factor. Results indicated a significant main effect of Time, *F*(1, 37) = 104.36, *p* < .001, η_p_^2^ = .74, which was qualified by a significant Condition × Time interaction, *F*(1, 37) = 5.26, *p* = .028 η_p_^2^ = .12 (see Figure 2). Paired samples *t* tests confirmed that participants in both conditions rated their affect as more negative from pre- to posttraining (abstract: *t*[18] = 8.22, *p* < .001, *d* = − 2.25, 95% CI [28.21, 47.58]; concrete: *t*[19] = 6.06, *p* < .001, *d* = − 1.62, 95% CI [15.71, 32.30]). A *t* test confirmed that the decrease in affect was greater in the abstract condition than in the concrete condition, *t*(37) = − 2.29, *p* = .028, *d* = 0.73, 95% CI [− 26.17, − 1.619].

### Effect of Processing Mode Training on the Development of Intrusive Memories and IES-R Scores

**Figure 3:**
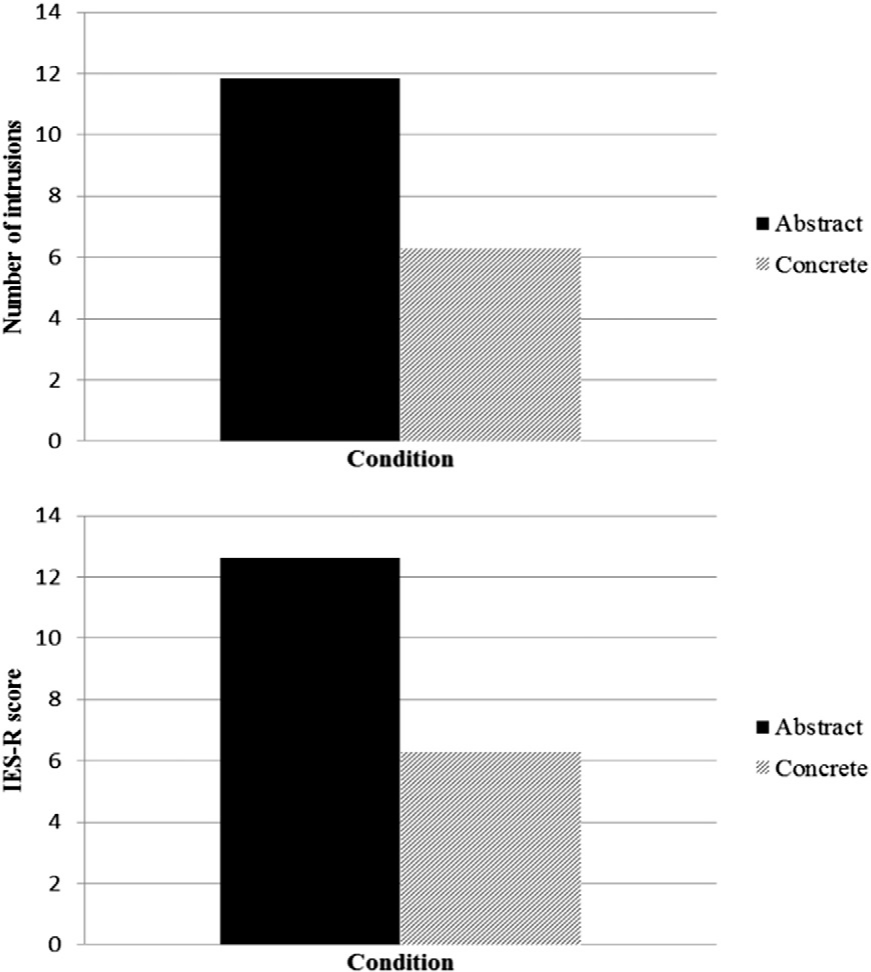
Mean number of intrusive memories and IES-R scores (with standard errors) by condition.

Of the whole sample, 47 participants (94%) reported at least one intrusive memory relating to the films. Figure 3 shows the number of intrusions and IES-R scores by condition. We conducted independent samples *t* tests to test our prediction that participants in the concrete condition would experience fewer intrusions during the following week and lower IES-R scores at 1-week follow-up. As predicted, participants in the concrete condition reported significantly fewer intrusive memories over the following week than those in the abstract condition, *t*(48) = 2.07, *p* = .044, *d* = 0.59, 95% CI [1.69, 4.53]. Likewise, participants in the concrete condition reported significantly lower IES-R scores than those in the abstract condition at 1-week follow-up, *t*(48) = 2.78, *p* = .009, *d* = 0.78, 95% CI [1.69, 10.95]*.*

We conducted bivariate correlational analyses to assess whether number of intrusions or IES-R scores were related to change in affect over the training session. These showed that there was no significant relationship between change in affect from pre- to posttraining and number of intrusive memories, *r* = − .20, *p* = .229, or between affect change and IES-R score, *r* = − .27, *p* = .095.

### Effect of Processing Mode Training on Emotional Reactivity

**Figures 4:**
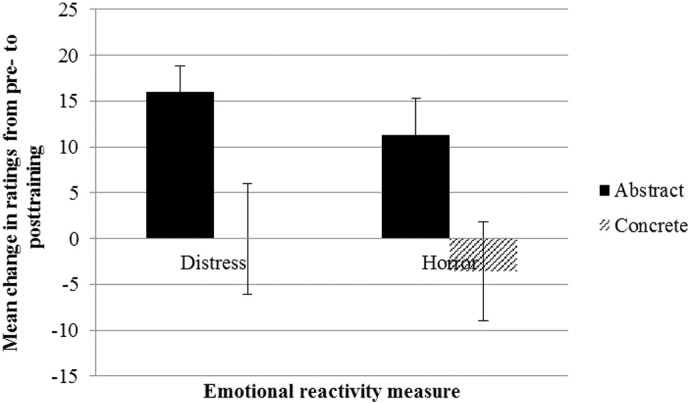
Mean change in distress and horror ratings of test films (with standard errors) by condition.

Figure 4 shows change scores in distress and horror ratings from pre- to posttraining by condition. To investigate the effect of training on emotional reactivity to the posttraining test film, we conducted a 2 (Condition) × 2 (Time) multivariate analysis of variance (MANOVA) on distress and horror ratings. Results indicated a significant multivariate Condition × Time interaction, *F*(1, 47) = 3.50, *p* = .038, η_p_^2^ = .13, which allowed us to interpret the univariate ANOVAs. Univariate ANOVAs revealed a significant Condition × Time interaction for distress ratings, *F* = (1, 48) = 5.95, *p* = .018, η_p_^2^ = .11, and horror ratings, *F* = (1, 48) = 5.03, *p* = .030, η_p_^2^ = .10. This suggests that concrete training led to less emotional reactivity to the posttraining test film relative to abstract training. Paired samples *t* tests comparing pre- and posttraining distress scores showed a significant increase in subjective distress ratings from pre- to posttraining test films in the abstract condition, *t*(25) = − 5.51, *p* < .001, *d* = 0.68, 95% CI [− 21.93, − 10.00], but no significant increase in the concrete condition *t*(23) = 0.00, *p* = 1.000, *d* = 0.00, 95% CI [− 12.51, 12.51]. Similarly, for horror ratings, a significant increase was found in the abstract condition, *t*(25) = − 2.87, *p* = .008, *d* = 0.44, 95% CI [− 19.50, − 3.20], compared with no significant increase in the concrete condition, *t*(23) = 0.65, *p* = .520, *d* = − 0.12, 95% CI [− 7.67, 14.76]. These findings suggest that abstract training led to both increased distress and horror reactions to the posttraining test film, whereas concrete training did not.

There was a significant correlation between change in affect and change in distress ratings, *r* = − .32, *p* = .047, with decreased affect from pre-to posttraining being associated with increased distress ratings. Since participants in the abstract condition showed a greater decrease in affect from pre- to posttraining than those in the concrete condition, we conducted a mixed model analysis of covariance (ANCOVA) to investigate whether increases in distress in the abstract condition could be attributed to decreases in affect rather than to mode of processing. However, when change in affect from pre- to posttraining was included as a covariate, the effect of condition on changes in distress ratings remained, *F*(1, 36) = 6.80, *p* = .013, η_p_^2^ = .16, indicating a significant increase in distress ratings in the abstract but not the concrete condition (abstract: *t*[25] = − 5.51, *p* < .001, *d* = 0.68, 95% CI [− 21.93, − 10.00], concrete: *t*[23] = 0.00, *p* = 1.000, *d* = 0.00, 95% CI [− 12.51, 12.51]). There was no significant correlation between change in affect and change in horror ratings, *r* = − .21, *p* = .191.

### Relationship Between Predictor Variables and Intrusive Memories

We conducted correlational analyses to explore relationships between predictor variables (trait rumination and dissociation, proneness to intrusive memories, and sleep difficulties) and primary outcome measures (number of intrusions and IES-R scores). None of these were significant apart from proneness to intrusive memories, which was significantly associated with the development of intrusive memories following the films. Since greater proneness to intrusive memories following negative events in everyday life was associated with a higher number of reported intrusions of the films, *r* = .32, *p* = .025, we conducted a univariate ANCOVA to investigate the effect of condition on intrusive memories while controlling for participants’ proneness to intrusive memories. There was a significant effect of condition on intrusions after controlling for the effect of proneness to intrusive memories, *F*(1, 47) = 4.40, *p* = .041, η_p_^2^ = .09, with fewer intrusive memories reported in the concrete compared with the abstract condition, *t*(48) = 2.07, *p* = .044, *d* = 0.59, 95% CI [1.69, 4.53].

## Discussion

This study investigated the effect of adopting an abstract or concrete mode of processing during exposure to analogue trauma and the subsequent development of intrusive memories. As predicted, individuals who were trained to adopt concrete information processing while watching the traumatic films reported fewer intrusive memories and lower PTSD symptom severity scores during the following week compared with individuals who were trained to adopt abstract information processing. Concrete information processing also led to less emotional reactivity (measured as distress and horror) to a posttraining film relative to abstract processing. Our results are consistent with the broader literature showing that processing mode influences responses to negative events (Watkins, 2008), and correlational research linking a ruminative, abstract style of processing after trauma to PTSD symptoms (e.g., El-Leithy et al., 2006, Michael et al., 2007). However, this appears to be the first study to show that processing mode (i.e., abstract vs. concrete) may be causally involved in the development of experiences such as intrusive memories, a hallmark feature of PTSD, and that undergoing training in concrete processing could protect against the development of intrusions. Furthermore, it supports models highlighting peritraumatic cognitive processing as a key factor influencing distress and the development of intrusive memories (e.g., Brewin et al., 1996, Ehlers and Clark, 2000). Since the current study focused on analogue trauma in a healthy, nontraumatized population, future research could investigate whether the current findings extend to real-life traumatic events and the subsequent development of PTSD symptoms.

There are different possible mechanisms by which concrete processing may be more adaptive than abstract processing. First, in line with existing cognitive models (e.g., Ehlers & Clark, 2000), concrete processing may lead to a more organized memory and more adaptive appraisals of the event. Halligan, Michael, Clark, and Ehlers (2003) found that memory disorganization and negative appraisals of a traumatic event predicted PTSD symptoms in assault survivors. By focusing attention on contextual details and the overall sequence of events, concrete processing may promote the formation of a coherent narrative, whereas abstract processing may disrupt this process. In addition, concrete processing may generate situation-specific appraisals whereas abstract processing may promote unhelpful, overgeneralized appraisals (Watkins, 2008). Future research could measure the coherence or accuracy of memories of the trauma films as well as assessing what kinds of appraisals participants formed of the analogue trauma. Second, in line with hypotheses about the beneficial effect of concrete relative to abstract processing more generally, a concrete style of thinking may facilitate emotional processing of distressing events (e.g., Stöber, 1998, Teasdale and Barnard, 1993), potentially preventing the development of anxious appraisals (e.g., Behar et al., 2012).

Since the abstract condition included instructions for participants to think about “Why these sorts of things happen,” “What it means about the world,” and “What if this was to happen to you or someone in your family?” one rival hypothesis to explain the current findings is that abstract training discouraged participants from processing the films in a self-referent way. Self-referent processing refers to organizing information in relation to the self: in relation to one’s present experience as well as to past experiences and future goals (Farb et al., 2007). Self-referent processing thus involves processing what is happening in the context of oneself and distinguishing one’s own experiences from what is being witnessed. Lack of self-referent processing has been associated with the development of PTSD symptoms, such as intrusive memories, in clinical and nonclinical samples (Evans et al., 2007, Laposa and Rector, 2012), presumably because when individuals fail to engage in self-referent processing, they are less likely to notice how the experiences they are witnessing are different from their own experiences. Future studies could include a measure of self-referent processing (e.g., Ehlers, 2002).

Proneness to intrusive memories of stressful or unpleasant events in everyday life was associated with greater intrusions of the trauma films, which is consistent with Davies and Clark (1998), who found that self-reported tendency to experience intrusive memories predicted intrusive memories of a traumatic film clip. It is unclear why some individuals would have a greater tendency to experience intrusive memories, although this may relate to individual differences in ways of processing negative events (e.g., adopting an abstract-ruminative style) or strategies used to regulate emotions in response to them. Future research could further investigate processing styles or emotion regulation strategies that may be linked to intrusive memories after negative events in everyday life. However, even after controlling for proneness to intrusive memories, a significant effect of condition remained, suggesting that even if one were vulnerable to experiencing intrusions, the likelihood of developing intrusive memories after analogue trauma could be reduced through training in concrete processing.

There are limitations that should be considered when drawing conclusions from this study. First, our study adopted an analogue design with a nonclinical sample, therefore we are unable to say whether these findings would generalize to real-life traumatic events. Second, since we did not include a no-training control condition, it is difficult to determine whether concrete training is superior to no training at all. However, because our hypotheses related to the effects of concrete compared with abstract training, our design fit the hypotheses to be tested and is consistent with the design of other studies seeking to compare abstract versus concrete information processing (e.g., Watkins, 2004, Watkins et al., 2008). Future research could include a no-training control condition to ascertain whether the effects of concrete processing are indeed superior to no training at all, as well as to abstract processing.

In conclusion, this study provides the first empirical indication that relative to processing analogue traumatic stimuli in abstract ways, processing them in concrete ways leads to fewer intrusive memories over the following week and lower emotional reactivity to a subsequent analogue trauma. Our study also indicates that individuals who self-reported proneness to intrusive memories in everyday life were more likely to develop intrusive memories in response to analogue trauma and that concrete processing may reduce vulnerability to developing intrusions in these individuals. These findings improve our understanding of the role of peritraumatic processing in the development of intrusive memories and PTSD symptoms. It would be important to assess the generalizability of the current findings to at-risk occupational groups who are regularly exposed to trauma, such as emergency service personnel and journalists in conflict zones, since this could inform the development of interventions aimed at protecting against intrusive memories and PTSD in such populations.

## Conflict of Interest Statement

The authors declare that there are no conflicts of interest.

## Figures and Tables

**Table 1:** Sample Characteristics and Means (With Standard Deviations) at the Initial Pretraining Assessment by Condition

Variable	Condition	
Abstract (*n* = 26)	Concrete (*n* = 25)
Gender	15 female	13 female
Age (years)	27.15 (9.11)	24.71 (5.35)
Drove frequently (at least once a week)	7 (26.9%)	6 (25%)
PHQ-9	1.88 (2.18)	2.17 (1.20)
STAI-T	30.15 (7.15)	32.58 (7.19)
Trauma checklist	1.38 (1.42)	1.33 (1.61)
IES-R (baseline)	5.15 (6.29)	6.54 (7.83)
PTQ	14.54 (9.47)	16.83 (11.26)
TDQs	6.92 (5.76)	6.75 (4.23)
ISI	5.31 (5.42)	4.38 (2.84)
PIMS	1.31 (1.16)	1.25 (1.03)
Baseline affect	75.25 (16.82)	72.75 (11.72)
Personal relevance of pretraining test film	15.00 (23.37)	14.17 (21.65)
Personal relevance of posttraining test film	35.38 (31.53)	27.71 (30.29)
Pretraining test film distress	49.81 (23.34)	45.21 (25.43)
Pretraining test film horror	47.12 (24.67)	41.46 (27.72)

*Note.* PHQ-9 = 9-item Personal Health Questionnaire; STAI-T = State Trait Anxiety Inventory-Trait version; IES-R = Impact of Events Scale-Revised; PTQ = Perseverative Thinking Questionnaire; TDQs = Trait Dissociation Questionnaire-short version; ISI = Insomnia Severity Index; PIMS = Proneness to Intrusive Memories Scale.

## Footnotes

2 Four participants in the concrete condition and seven participants in the abstract condition did not complete this measure before and after the films. Therefore, the total sample included in analyses relating to this variable only is *N* = 39 (rather than *N* = 50).
